# Victimization and PTSD-like states in an Icelandic youth probability sample

**DOI:** 10.1186/1471-244X-7-51

**Published:** 2007-10-01

**Authors:** Íris Bödvarsdóttir, Ask Elklit

**Affiliations:** 1 The National Diagnostic and Counselling Centre, Digranesvegi 5, 200 Kópavogur, Iceland; 2 Department of Psychology, University of Aarhus, Jens Chr. Skous Vej 4, DK-8000 Aarhus C, Denmark

## Abstract

**Background:**

Although adolescence in many cases is a period of rebellion and experimentation with new behaviors and roles, the exposure of adolescents to life-threatening and violent events has rarely been investigated in national probability studies using a broad range of events.

**Methods:**

In an Icelandic national representative sample of 206 9th-grade students (mean = 14.5 years), the prevalence of 20 potentially traumatic events and negative life events was reported, along with the psychological impact of these events.

**Results:**

Seventy-four percent of the girls and 79 percent of the boys were exposed to at least one event. The most common events were the death of a family member, threat of violence, and traffic accidents. The estimated lifetime prevalence of posttraumatic stress disorder-like states (PTSD; DSM-IV, APA, 1994 [1]) was 16 percent, whereas another 12 percent reached a sub-clinical level of PTSD-like states (missing the full diagnosis with one symptom). Following exposure, girls suffered from PTSD-like states almost twice as often as boys. Gender, mothers' education, and single-parenthood were associated with specific events. The odds ratios and 95% CI for PTSD-like states given a specific event are reported. Being exposed to multiple potentially traumatic events was associated with an increase in PTSD-like states.

**Conclusion:**

The findings indicate substantial mental health problems in adolescents that are associated with various types of potentially traumatic exposure.

## Background

Epidemiological studies on a broad range of potentially traumatic events and posttraumatic stress disorder-like states (PTSD) in adolescents that build on national probability samples are quite rare in contrast to the considerable number of convenience sampling studies focusing on children and adolescents exposed to specific events. In a national probability sample, Elklit [2] studied 390 Danish 8^th ^graders age 12–15 (Mean = 14.0 years, SD .37); and found that 78 percent of the males and 87 percent of the females had been exposed to at least one potentially traumatic event. The estimated lifetime prevalence of PTSD (measured by the HTQ-IV – see below) in the sample was 9.0 percent, whereas another 14.1 percent reached a sub-clinical level of PTSD. Following exposure, females suffered from PTSD twice as often as males. The most common events were the death of a family member, threat of violence, and serious accidents. The most distressing subjective events were rape, suicide attempts, death in the family, serious illness, and childhood abuse. Gender, parents' education, and living with a single parent were associated with specific events. Being exposed to multiple potentially traumatic events was associated with an increase in PTSD.

Domainskaite-Gota & Elklit: (Victimization and PTSD in a Lithuanian national youth probability sample, submitted) studied a national probability sample of 183 9^th ^grade Lithuanian adolescents (M = 15.1 years; SD .61; range 13–17 years) and found that 81 percent of the males and 80 percent of the females had been exposed to at least one potentially traumatic event. Most frequent events were threats of physical assault, near-drowning experiences, death of someone close, and robbery/theft. Estimated lifetime prevalence of PTSD (measured by the HTQ-IV – see below) was 6.1% with 12.2% reaching a sub-clinical level of PTSD. Female gender, living with a single parent, direct and indirect exposure to potentially traumatic events, number of events, and more recent exposure (< 1 year) predicted higher PTSD levels.

In an Israeli convenience youth sample of 494 9th and 10th grade students (M = 15.6 years; SD .72; range 14–18). Andersen, Elklit & Lasgaard: (The prevalence and impact of traumatic events in Israeli youth, submitted) studied 20 potentially traumatizing events along with their psychological impact on the Trauma Symptom Checklist (TSC). Eighty-five percent of the students had been exposed to at least one potentially traumatic event. The most common events recorded were almost being injured or killed, war, and violent assault. Socio-demographic variables were found to be associated with specific events. The total number of events was associated with symptomatology, and the presence of recent exposure was associated with somatization and total symptom score. The events most strongly associated with symptom level were humiliation or persecution, attempted suicide, and childhood neglect.

As part of a general population study Costello et al. [3] interviewed 1420 children and adolescents aged 9–13, living in a poor rural part of North Carolina, about 30 potentially traumatic events. By age 16 one quarter had experienced at least one high magnitude event, with 6% experiencing a high magnitude event within 3 months prior to the study. One third experienced a low magnitude event within 3 months prior to the study.

In a longitudinal study of 384 18-year-old working-class US adolescents, Giaconia et al. [4] found that 43% had experienced at least one traumatic event (as defined by the DSM-III-R [5]) out of a list of 11 stressful events. Fifteen percent of the exposed youths or 6 percent of the total sample developed PTSD assessed by means of a structured clinical interview. Youths who had experienced potentially traumatic events demonstrated behavioural emotional problems, interpersonal problems, academic failure, suicidal behaviour, and physical health problems by the age of 18 – regardless of whether or not they developed PTSD. Eighty percent of the adolescents with PTSD met criteria for at least one additional disorder, and 40 percent had two or more lifetime disorders. Giaconia et al. [4] stressed that those who work with adolescents should not underestimate the potential of a wide range of stressful events to provoke PTSD symptoms and associated problems later in the adolescents' lives – including events that do not involve direct violence or physical harm. They also emphasize the importance of investigating other factors such as sample characteristics and family environment.

Joseph et al. [6] examined the prevalence of 12 negative life events in a convenience sample of 427 English adolescents aged 11 to 16 (Mean age for boys = 13.7, SD = 1.5; mean age for girls = 13.8, SD = 1.3) and found that 84% endorsed at least one event, the most common being a threat to the life of a family member or friend (accident, injury or illness). The impact of the aversive events was measured by the Impact of Event Scale.

A criminological study [7] of 1,270 Danish 8th-grade students from three different regions of the country found that 44 percent had experienced a theft, 16 percent had been beaten, and 36 percent had been threatened with violence within a period of 12 months. Twenty-six percent of the students in the Balvig study came from a split home. A Swedish study [8] of a national sample of 17-year-old adolescents found that 2.3 percent of the boys and 7.1 percent of the girls had experienced sexual abuse. The corresponding data for abusive intercourse were 1.2 percent for boys and 3.1 percent for girls, and for suicide attempts (and self-mutilating acts), were 5.9 percent for boys and 11.4 percent for girls.

All the reviewed studies (but the Giaconia et al. study that used structured clinical interviews) assessed the events retrospectively by self-report. The reviewed studies give ample evidence that youth in several countries are exposed to a considerable amount of potentially traumatic and distressing life events and that the PTSD-like state prevalences are comparable to what is found in adult studies. National probability studies provide authorities with reliable estimates of service needs that are missing in mental health planning.

Hill and Jones [9] examined children's and parents' perceptions of children's exposure to community violence and found a remarkable discrepancy between the two groups. The children reported exposure to much more violence than their parents were aware of. The parents' lack of awareness may increase the risk of inadequate guidance of the young in situations where they might need the most guidance. The same could be said about professional guidance of children and adolescents. To meet the requirement of optimal professional guidance, it is important to examine the prevalence of different types of victimization and distressing living conditions. The current study was designed to provide epidemiological information about exposure to potentially traumatic events and life events together with PTSD-like states in an Icelandic national probability study. The purpose was threefold: 1) to examine the relationship among the experiences of potemtial traumatic events, life events, socio-demographic variables, and PTSD-like states; 2) to estimate the lifetime prevalence of potentially traumatic events, life events, and PTSD-like states, overall, and according to gender; and 3) to assess the subjective distress of a number of potentially traumatic events and life events.

## Methods

### Study design and sample

The data in this study were collected from a questionnaire survey with a national representative probability sample of 206 Icelandic 9^th ^graders aged 13 to 15. The sample was geographically stratified by four regions, with sample allocation proportionate to the national population distribution. In Iceland, five schools in each region that taught the grade level students were chosen by permutations (random numbers) and approached.

Twenty schools that taught 9th-grade students were approached. Fifteen schools accepted and participated. The primacy of the initials of the head teachers decided which class was chosen, in case there was more than one 9th grade class in the school. The number of students in the class varied between 9 and 26 students (average 19.1). All students present except one completed the questionnaires.

The study was introduced through: A) a letter to the headmasters explaining the selection procedure; B) a letter to the head teacher describing the introduction, monitoring, support, and confidentiality procedures – i.e., the sealing of the return envelopes in front of the pupils; and C) a letter to each pupil explaining the purpose, the confidentiality, the option of not participating, and the collecting procedures. The parents' written consent was a prerequisite for participating. The study was approved by the Icelandic scientific committee.

### Instruments

The first part of the questionnaire contained questions about gender, age, parents' education, and living arrangements (living with one parent, two parents, or others such as grandparents or within an institution). Parents' education was chosen as a crude measure for the socioeconomic situation. More detailed demographic information was not asked because other studies, e.g. [7], have shown that adolescents' knowledge of parents' income and occupational status is not very reliable.

In the last part of the questionnaire, the students were asked about their exposure to 20 potentially traumatic events and life events (see Table 1). Each question could be answered according to direct exposure or indirect exposure (i.e. witnessing an event or a person close to them experiencing an event). The list of events was selected from scientific literature and clinical experience, covering possible life-threatening experiences and distressing family conditions such as neglect, abuse, and absence of a parent.

**Table 1:** Potential Trauma Events and Life Events According to Exposure and Gender

		Direct exposure in percentage			Indirect exposure in percentage		
Event		females (n = 103)	males (n = 100)	all ^a) ^* (= 206)	females (n = 103)	males (n = 100)	all ^a) ^* (= 206)

1	Traffic accident	22.3	32.0	27.1	46.6	52.0	49.0
2	Other serious accidents	10.7	12.0	11.1	32.0	34.0	33.0
3	Physical assault	6.8	9.0	7.8	15.5	17.0	16.0
4	Rape	5.8	1.0	3.3	11.7	7.0	9.2
5	Witnessed other people injured or killed	6.8	5.0	5.8	20.4	23.0	21.8
6	Came close to being injured or killed	4.9	13.0	8.7^1) ^(4.21)	12.6	11.0	12.1
7	Threats of violence	17.5	39.0	27.6^2) ^(9.47)	16.5	17.0	17.0
8	Near-drowning	15.5	26.0	20.9	7.8	9.0	8.3
9	Attempted suicide	14.6	6.0	10.2^1) ^(4.05)	11.7	13.0	12.1
10	Robbery/theft	18.4	19.0	18.4	19.4	25.0	22.3
11	Pregnancy/abortion	4.9	0.0	2.5	9.7	9.0	9.4
12	Serious illness	1.9	8.0	4.8^2) ^(4.02)	51.5	32.0	41.7^2) ^(8.13)
13	Death of someone close	44.7	41.0	42.7	44.7	37.0	40.8
14	Divorce	19.4	21.0	20.4	19.4	14.0	17.0
15	Sexual abuse	5.8	2.0	3.9	6.8	7.0	6.8
16	Physical abuse	3.9	2.0	2.9	6.8	4.0	5.3
17	Severe childhood neglect	3.9	2.0	2.9	10.7	5.0	7.8
18	Humiliation or persecution (bullying)	23.3	23.0	23.3	12.6	18.0	15.0
19	Absence of a parent	8.7	3.0	5.8	7.8	6.0	6.8
20	Miscellaneous	12.6	4.0	8.3	4.9	2.0	3.4

^a) ^Three did not state their gender. * F-ratio values in parentheses. p <^1)^.05 ^2)^.005

Sexual abuse was defined as sexual exploitation in a close relationship by an older relative or another close person, but the study did not distinguish between intra- and extra-family perpetrators. Rape was defined as forced intercourse or sex. Abortion and pregnancy were lumped together in the questionnaire, because of very low occurrence of born babies and because of a desire to cover the situation where a female is pregnant and has not yet decided to have an abortion. The number of babies in Iceland born by 15 year-old or younger mothers is extremely low. We expect practically all pregnancy cases to be represented by abortions, but we did not want to signal that a pregnancy could not be a happy situation. Suicide attempt is often conceived as a result of mounting inner distress, but it can also be seen as an event in its own right choosing death and experiencing injuries, intensive care unit treatment and often stigmatization. Being persecuted and bullied by peers or adults can sometimes take the form of reduction of the victim's physical integrity and autonomy. There were two questions in the study about parenthood: one about divorce, and the other about absence of a parent. These questions were meant to identify situations when divorce is not formal or when one parent is absent from the household because of work, a family crisis, or other obligations. No psychometric data are yet available on the event measure.

The Harvard Trauma Questionnaire-Part IV (HTQ) [10] was used to estimate the occurrence of PTSD-like states at the time of the event. When filling in the HTQ, pupils were asked to write down the event most distressing to them and to keep that in mind when answering. The HTQ contains the 17 PTSD symptoms included in the DSM-IV [1]. The HTQ-Part IV has been used extensively in the Nordic countries and permits an assessment of whether or not a person suffers from PTSD-like state [11]. It is also a measure of the intensity of the three core symptom groups (re-experiencing, avoidance, and hyper-arousal) of a PTSD-like state. The items are scored on a four-point Likert scale (1 = not present, 4 = very often present). An item score must be ≥ 3 to count as a symptom for a quasi diagnosis. A sub-clinical level of a PTSD-like state is gained if the respondent meets two out of three criteria and misses the last criterion by only one symptom. The latter does not apply to the intrusion subscale, which must be reached. The subscales are scored separately. The internal consistency in this scale was found to be good: Cronbach's alpha = .97 for the 17 PTSD questions and .79, .84, and .85 for the re-experiencing, avoidance, and hyperarousal subscales, respectively. The inter-item coefficients for the subscales were .51, .44, and .53 respectively, indicating good discriminatory power [12]. Mollica et al. [10] found an 88% concordance between interview based and HTQ self-report based diagnosis of PTSD.

The gender distribution was 50 percent girls and 49 percent boys (3 did not report their gender). Seventy-six percent of the pupils lived with both parents, 23 percent lived with one parent, and 1 percent had other arrangements. The difference between the parents' education was significant (t = 2,24; p < .05 – fathers vs. mothers: Primary school 24% vs. 31%, high school 18% vs. 29%, college 33% vs. 8%, and university 26% vs. 31% respectively). There were no significant differences between the various regions of the country in level of participation.

## Results

The most common event recorded (Table 1) was death of a family member (42.7 percent), followed by threats of violence (27.6 percent), traffic accidents (27.1 percent), humiliation or persecution by others (23.3 percent), and near-drowning (20.9 percent). Least prevalent was sexual abuse (3.9 percent), rape (3.3 percent), physical abuse (2.9 percent), severe childhood neglect (2.9 percent), and pregnancy/abortion (2.5 percent). The prevalence of indirect exposure to potentially traumatic events was generally higher than direct exposure. The most noticeable exceptions were humiliation or persecution by others, threat of being beaten, and near-drowning, all of which may be events of a more private than a public nature.

Table 1 also revealed gender differences. Boys more often than girls, had been close to injury, met received threats of being beaten, and experienced serious illness. Girls were more prone to attempt suicide than boys. Girls, via indirect exposure, had been witness to serious illness more often than boys.

The number of serious, potential life threatening and distressing events was considerable. The average number of direct events per pupil was 2.6 (percentage who experienced one event = 21 %, two events = 17 %, three events = 11 %, four events = 10 %, five events or more = 18%), and the average number of indirect events per pupil was 3.5 (percentage who witnessed one event = 14 %, two events = 10 %, three events = 15 %, four events = 10 %, five events or more = 30 %). Rape, sexual abuse, childhood neglect, and physical abuse were strongly interrelated (all χ^2 ^> 40.0; all df = 1; all p's < .0005).

In Table 2, the odds ratios for each event are provided. Rape, absence of a parent, bullying, physical assault, attempted suicide, and death of someone close were associated with *OR'*s > 2.0, while childhood abuse and neglect, pregnancy/abortion, having been close to injury and witnessing other people becoming injured or killed had *OR*'s < .50.

**Table 2:** Odds Ratios and 95% CI Intervals for the Potential Trauma Events and Life Events in Predicting a PTSD-like State

		95% CI for Exp (B)		
Event		OR	Lower	Upper

1	Traffic accident	1.42	.50	4.08
2	Other serious accidents	.66	.14	3.11
3	Physical assault	3.65	.64	20.64
4	Rape	31.0*	1.14	845.20
5	Witnessed other people injured or killed	.44	.04	5.46
6	Came close to being injured or killed	.36	.05	2.46
7	Threats of violence	1.31	.78	2.18
8	Near-drowning	1.50	.53	4.25
9	Attempted suicide	2.69	.74	9.72
10	Robbery/theft	.56	.14	2.22
11	Pregnancy/abortion	.47	.01	26.72
12	Serious illness	.93	.07	13.02
13	Death of someone close	2.12	.85	5.28
14	Divorce	.42	.10	1.77
15	Sexual abuse	.31	.00	38.74
16	Physical abuse	.01	.00	9.12
17	Severe childhood neglect	.49	.01	19.19
18	Humiliation or persecution (bullying)	4.12*	1.54	11.04
19	Absence of a parent	11.79*	1.43	97.42
20	Miscellaneous	2.34	.62	9.53

Note: * = p < .05

Mothers' primary school educational level meant increased risk for divorce. The mothers' education was also associated with traffic accidents and other serious accidents, robbery/theft, and divorce (all χ^2 ^> 8.3; all df = 3; all p's < .05) in that way that college education constituted a risk factor. Fathers' and mothers' university education was only a risk factor with the event of coming close to being injured or killed; mothers' university education was also associated with increased risk of robbery/theft. Living in a single-parent household was – besides from divorce and absence of a parent – associated with several events (all χ^2 ^> 5.2; all df = 1; all p's < .05): more traffic and other serious accidents, more threats of beating, robbery/theft and physical assault (the latter: χ^2 ^= 3.6; df = 1; p = .06).

Coming from a single-parent household was associated with total number of direct and indirect exposure events (both F's _(1,205) _> 113.6; both p's < .002). Gender, age, and parents' education were not associated with total number of events with the exception of mothers' education and indirect exposure (F _(1,205) _= 43.6; p < .01). Of the 161 students who had reported one potentially traumatic event and given full information on the HTQ, 12 boys and 21 girls fulfilled all the criteria for a PTSD-like state at the time of the event, corresponding to 16 % of the total sample and 20.5 % of the exposed sample. Eleven boys and 13 girls, or 12 % of the total sample constituted a sub-clinical group, missing the PTSD-like state by one symptom in the C and D symptom clusters according to the DSM-IV. The difference between gender in the rate of PTSD-like states was significant (F _(1,156) _= 3.9; p <. 05). Age, parents' education and single-parent household were not associated with PTSD-like state rates.

PTSD-like state rates were associated with number of direct exposure events (r = .25; p = .001) and number of indirect exposure events (r = .21; p = .01). Overall, there was a considerable gender difference in that girls were more prone to develop PTSD-like state or sub-clinical PTSD-like state than boys after most event types, including: death in the family, threats of violence, physical assault, witnessing injury, sexual abuse, rape, and abortion. The only exceptions were divorce and serious illness where boys were more likely to develop a PTSD-like state or a sub-clinical PTSD-like state. One should note that for several events there are a modest number of endorsements.

## Discussion

The present study revealed, in line with other studies from other countries [2,6,9], that adolescents had experienced a large number of potentially traumatic events. Death of someone close and threats of physical violence were the most common events experienced, corresponding to findings from Denmark and Lithuania. There were surprisingly few gender differences: Boys were more often threatened with violence, were closer to getting injured, and experienced serious illness more often than girls, while girls reported more suicide attempts. The prevalence of suicide attempts is similar in level for both genders to that found in the Swedish national probability study by Edgardh & Ormstad [8]. There were no overall gender differences in total exposure. Two life event questions concerned parenthood: one divorce and the other absence of a parent. The latter question was meant to identify situations when divorce was not formal, when parents were separated, or when one parent was absent from the household because of work, family crisis, or other obligations. When looking at the divorce item, there was no significant difference between genders, but with the absence item, girls reported absence of a parent almost three times more than boys – similar findings are reported by Elklit [2] and Costello et al. [3]. This finding might be due to factual circumstances that fathers are more reluctant to leave their families if there is a boy present. Another explanation could be that girls are more attentive and/or vulnerable to parent absence.

In a longitudinal study of risk and resilience factors Werner and Smith [13] found that level of education was supportive in a gender specific way; mothers' education had a protective effect for boys, and fathers' education had the same effect for girls. However, the Werner and Smith finding was not supported by the data in the present study. We found an association between especially better-educated mothers and a few potentially traumatic events that indicate a function of better access to motorized vehicles and living standards that make their homes more attractive to burglars.

The present study found high OR's for PTSD-like states after a number of potentially traumatic events and for two life events (bullying and absence of a parent). The OR's for PTSD-like states associated with the absence of a parent is in contrast to the OR's concerning divorce; the difference might be explained by a higher level of conflict between parents and a higher level of uncertainty in the adolescent. Surprisingly, childhood abuse and neglect were not associated with high OR's for PTSD-like states. We do not have an explanation for that finding.

Breslau et al. [14] found that females had a much higher risk of developing PTSD compared to males when socio-demographic factors and type of trauma were controlled for. This overrepresentation of females is similar to that found in the present study and in many other studies.

PTSD-like state rates were high for females for rape, suicide, physical assault, traffic accidents, threats of violence, bullying, and parent absence. PTSD-like state rates were high for males in relation to threats of violence, bullying, divorce, and parent absence. One should notice that the PTSD-like state appears in relation to the DSM-IV A1 stressor criterion and not in relation to life events. One may speculate that parental conflict in many cases may be so dramatic that many children experience the conflict as threatening to their basic security. The same might be the case in relation to threats of violence and bullying, which many adults might tend to underestimate. Joseph et al. [6] found parental separation and divorce to be associated with high levels of post-traumatic stress for both genders. They also found physical assault to be a much higher stressor for females than for males.

Some events are more likely to produce negative effects other than PTSD. The knowledge we have in various areas is unevenly distributed and concentrated on divorce, sexual abuse, and childhood neglect. In an Icelandic study of young adults whose parents divorced when they were children, Jónsson et al. [15] found that the main effects seemed to be a negative emotional stability and a less stable relationship pattern. Boney-McCoy and Finkelhor [16] found in their study of children and adolescents aged 10 to 16 that a third of the group had experienced violence and that sexual assaults were associated with an increase in symptoms. The potential seriousness of trauma in areas other than PTSD underscores the need for examining their effects more systematically during the critical developmental period of adolescence.

Some prevalent stressful events (traffic accidents, near-drowning, and divorce/absence of a parent) deserve to receive more attention from a traumatic stress point of view, whereas the study of other events, such as potential and physical violence, might benefit from supplementary knowledge of the co-occurrence of other potentially traumatic events. In line with the Pelcovitz et al. [17] study, an increase in exposure to stressful events was associated with an increase in PTSD-like states.

## Conclusion

In conclusion, there is increasing evidence to suggest that potentially traumatic events are as much a part of adolescence as they are part of adulthood. Boys and girls were found to be equal in their exposure to potentially traumatic events, but girls were found to be more vulnerable. Vulnerability was also connected to single parenthood due perhaps to a lack of parental supervision and stable role models, and/or to parental conflict. The endorsement of events was not randomly distributed, as four severe forms of abuse and neglect were found to be strongly associated. Despite methodological differences the present study is in agreement with previous studies investigating the mental health problems adolescents present when exposed to potentially traumatic events.

The present study has several limitations. The study relies on students' self-report with the inherent problems of potential response bias from for example their willingness to report accurately, their ability to be factual, or their willingness to remember painful events. Willis & Gonzalez [18] strongly advocate the use of event lists when assessing a vulnerable population, arguing that recognition is a much less distressful endeavour than recall when reporting emotionally painful situations. On the other hand, there might also be less of a memory bias, as some events may be more recent compared to a similar study using an adult population. In addition, the anonymity of the classroom could for some make reporting easier than in an interview study. The construction of the questionnaire placed the list of events almost at the end to prevent a biased attitude toward the trauma issue. The event questionnaire has not been validated, but in other national probability studies where it has been used [2] the degree of detail in items appears to be appropriate. In addition, the endorsement of specific events corresponds to findings from other studies. In a validation study of a trauma event questionnaire Kubany et al. [19] found very good concordance between interview data and questionnaire data when asking about trauma events. Due to the design of the study, there was no way of reporting whether a certain event had occurred more than once.

The studies above, as well as the present study, support the findings that adolescence is a risk period with a considerable exposure to stressful events. Most studies of adolescents in relation to trauma have focused on violence, but there seems to be ample evidence to suggest that events in addition to violence are important determinants of traumatization and that by focusing solely on violent acts and threats of violence one might miss a more comprehensive understanding of the totality of distressing events influencing development in adolescence. As the number of adolescents exposed to potentially traumatic events is considerable and as one in six at one point in their lives suffer from PTSD-like states, it is important that mental health professionals learn to identify adolescents at risk and offer intervention where needed. Health and educational personnel may want to integrate a standard procedure for obtaining information about stressful events from adolescents as part of the assessment and the planning of interventions. Such routine procedures may result in a broader and more effective intervention program for this age group.

## Competing interests

The authors declare that they have no competing interests.

## Authors' contributions

AE conceived of the study. Both authors participated in its design and coordination. IB collected the data. AE drafted the manuscript and performed the statistical analysis. Both authors read and approved the final manuscript.

## Pre-publication history

The pre-publication history for this paper can be accessed here:


